# A pilot model of a public–private partnership for implementation of a coronavirus disease 2019 (COVID-19) diagnostic testing program to facilitate a safe school reopening

**DOI:** 10.1017/ash.2021.249

**Published:** 2022-01-12

**Authors:** Westyn Branch-Elliman, Polly van den Berg, Sara W. Dong, Andrew K. Kapoor, Elisabeth A. Merchant, Elissa M. Schechter-Perkins

**Affiliations:** 1 Veterans’ Affairs (VA) Boston Center for Healthcare Organization and Implementation Research (CHOIR), Boston, Massachusetts; 2 Section of Infectious Diseases, Department of Medicine, VA Boston Healthcare System, Boston, Massachusetts; 3 Department of Medicine, Harvard Medical School, Boston, Massachusetts; 4 Section of Infectious Diseases, Department of Medicine, Beth Israel Deaconess Medical Center, Boston, Massachusetts; 5 Department of Emergency Medicine, Boston University School of Medicine and Boston Medical Center, Boston, Massachusetts

## Abstract

**Objective:**

We developed an implementation plan to integrate diagnostic testing for coronavirus disease 2019 (COVID-19) into a public school system. Implementation barriers were identified and strategies were mapped to overcome them.

**Design:**

A COVID-19 diagnostic testing program leveraging a public–private partnership was developed for a public school system.

**Setting:**

A suburban school district and a local hospital during the 2020–2021 academic year.

**Methods:**

Using Consolidated Framework for Implementation Research (CFIR) constructs and evidenced-based implementation strategies, the program was designed as a “closed system” and was adapted based on stakeholder feedback. Implementation barriers and facilitators were identified and mapped to CFIR constructs to provide insights into factors influencing program adoption.

**Results:**

Preimplementation stages of engagement, feasibility, and readiness planning were completed. The program did not progress to implementation due to multiple factors, including changes in school leadership (inner setting and process-level constructs), improved access to outside testing, and lack of an existing paradigm for in-school testing (external constructs). Limited support from key stakeholders and opinion leaders was also a barrier (process-level construct).

**Conclusions:**

Although this locally initiated program did not progress beyond the preimplementation stage, the processes developed and barriers identified may be useful to inform planning efforts in other testing programs within public school systems. Future programs may consider incorporating multiplex diagnostic testing for influenza in addition to COVID-19. With relaxation of infection control measures, the prevalence of other respiratory viruses will increase. Actionable results will be needed to inform decisions about closures and quarantines.

Elementary and secondary schools nationwide continue to grapple with how to integrate infection control strategies into primary and secondary school settings while prioritizing in-person instruction and minimizing time out of the classroom during the coronavirus disease 2019 (COVID-19) pandemic.^1^ Testing is an important mitigation strategy, and one that can facilitate learning; however, how to integrate COVID-19 testing, either screening or diagnostic, into primary and secondary school settings remains a critical question. Updated CDC guidance for the 2020–2021 school year addresses screening testing but not diagnostic programs.^2^

COVID-19 screening programs, such as the pooled antigen saliva testing program available in Massachusetts,^3–5^ identify presymptomatic and asymptomatic cases, provide information about in-school prevalence of disease, and may be used to evaluate in-school transmission. Diagnostic testing is applied to individuals with symptoms (by definition, a group with a higher pre-test probability of disease) to confirm the presence or absence of an active infection.

Within the United States, including the start of the 2020–2021 academic school year, COVID-19 diagnostic testing was obstructed by shortages at all stages of the supply chain, including limited availability of supplies, testing machines, and inadequate venues capable of performing testing.^6,7^ Turnaround times were long, appointments were difficult to arrange, and receipt of results often required navigation of multiple different websites. These factors created many challenges for school operations; lack of information meant long unnecessary quarantines and classroom or school closures. Furthermore, mechanisms for reporting results back to schools were deficient. The lack of closed-loop communication and an integrated tracking system was a particularly daunting challenge for tracking test results of school staff members, who may not live and work in the same district.

To address these challenges, our group developed a pilot proposal for integrating a COVID-19 diagnostic testing program into a public school system, considering different constructs within the Consolidated Framework for Implementation Research (CFIR).^8^ The testing model was developed following an implementation road map.^9^ It leveraged several evidence-based implementation strategies,^10^ and it was designed as a “closed system” to limit the potential for lost or missing results.

## Methods

### Setting

A pre-kindergarten through grade 12 public school district in Massachusetts and a private hospital are located in the same community; the community hospital receives a tax exemption in exchange for providing services to the town. The project development occurred primarily during the summer of 2020, and plans were being made to open in a hybrid learning model for the 2020–2021 academic year. Prior to reopening for the 2020–2021 school year, the local public school district’s leadership convened a Return to School Task Force, made up of community volunteers, which included a subcommittee on health. That subcommittee was made up of school personnel and other members of the community who had expertise in public health. This subcommittee was tasked with developing and proposing infection prevention strategies, which were presented to school leadership and the elected school committee for possible implementation. This health subcommittee identified access to testing as a major barrier for safe school operations and thus sought to identify strategies for improving testing access for students and staff.

### Program aims and key elements

The goals of this project were to design and implement a COVID-19 testing program embedded within a public school system. At the time the project was being developed, no similar programs were available.

Specific goals of the testing program were to improve access to testing with rapid turnaround and rapid availability of results. These goals were chosen (1) to limit spread of SARS-CoV-2 within school settings, (2) to limit time in quarantine and classroom closures due to delays in availability of test results, and (3) to ensure that test results were relayed quickly to the school system, so that decisions could be made about the need for quarantine or returning to school with a negative result. Given these goals, the entire test reporting system was designed to be embedded within the online education platform supported by the district. This ensured a record of a testing request, standard and organized collection of necessary data, and a central repository of key forms (eg, consent for testing forms) to streamline the process. Several key stakeholder groups were identified and engaged in discussions and planning. These included stakeholders from the school system (teachers, school nurses and front office staff, and school leadership including the school superintendent and elected representatives of the town school committee), parental volunteers on the reopening committee (including physicians and public health experts), hospital stakeholders (hospital leadership, laboratory personnel, and members of the compliance and billing office), and other local stakeholders, including members of the town government and local board of health.

The processes of program development are described here, and implementation frameworks (Stages of Implementation Completion and the CFIR) were used to characterize the stages of the process and to identify factors responsible for failure to progress beyond the preimplementation stage. The Stages of Implementation Completion is a process framework that highlights that the implementation process progresses through several stages, including preimplementation, implementation, and sustainment.^11,12^ The CFIR is a determinant framework,^8,13^ meaning that it can be used to evaluate factors (barriers and facilitators) that are associated with the ultimate success or failure of implementation. The 4 major CFIR constructs are organized into smaller domains: (1) characteristics of the intervention (eg, adaptability, complexity, cost), (2) the outer setting (eg, needs and resources, external policies and incentives), (3) the inner setting (eg, culture, climate, readiness for implementation, structural characteristics), and (4) the specifics of the implementation process (eg, engaging opinion leaders and champions). These domains were used to identify reasons why the program failed to progress past the preimplementation stage.

## Results

### Stages of implementation

The proposed program moved through the 3 stages of preimplementation (engagement, feasibility, and readiness planning) but did not progress to full implementation.^11,12^ Implementation strategies used during various stages of the implementation process, including details of how they were integrated into the plan, are presented in Table 1.

**Table 1.** Implementation Strategies Used During Stages of the Design Process

Implementation Strategies	Integration into Testing Program
**Preimplementation phase (completed)**	
Access new funding	Town-wide fund-raising campaign to support equitable availability of testing
Assess for readiness and identify barriers and facilitators	Ongoing discussions with key stakeholders, with feedback about barriers and acceptability of strategies
Build a coalition	Identify stakeholders and champions throughout the district
Develop educational materials, capture and share knowledge, distribute educational materials	Develop and disseminate an educational program
Change record systems	Develop a system for collecting and reporting of results. Integrate informed consent process within existing infrastructure.
Conduct educational meetings and conduct educational outreach	Provide educational sessions to key stakeholders in the community
Conduct local needs assessment	Identify and define the need for testing within schools
Develop partnerships	Leverage existing relationships with educational and hospital leadership
**Implementation phase (planned for future)**	
Facilitate relay of clinical data to providers	Create a new system for ordering testing within the schools, with direct reporting back to the schools to ensure closed-loop communication
Facilitation	Assistance and feedback form the return to school group, which included parental volunteers with expertise in key content areas
Identify and prepare champions	Find individuals dedicated to implementation of the testing program and overcoming indifference and challenge to the program
Identify early adopters	Find staff and students who utilized the testing program and learn from their experiences
Inform local opinion leaders	Inform influential school leadership about the testing program in hopes they will support staff and students to utilize the program
Intervene with consumers to enhance uptake and adherence	Develop strategies to encourage use of testing program
Make billing easier	Ensure that billing for test is simple
Prepare consumers to be active participants	Enable staff and students/families to ask questions & receive information about the testing program
Promote adaptability	Identify ways to tailor the program and foster sustainability
Promote network weaving	Build upon existing relationships and networks within and outside of the district to promote collaboration in implementation of the testing program
Provide local technical assistance	Deliver technical assistance related to implementation issues
Provide ongoing consultation	Consultation with local experts
Purposely re-examine the implementation	Monitor the program and adjust implementation
**Sustainment (planned for future)**	
Stage implementation scale-up	Pilot model testing program with goal expansion to testing asymptomatic members of the index case followed by periodic routine surveillance screening
Use advisory boards and workgroups	Create a diverse group of stakeholders for input and advice
Use mass media	Publicize the testing pilot program

### Iterative process development

#### Evaluation of successful testing programs in other settings

Prior to designing an operational plan, volunteer physicians on the health subcommittee of the town reopening task force reached out to several groups that already had established testing plans, including a local private college and private K–12 school, a private company that provided testing services in other towns, and the local board of health, to identify potential testing strategies that might be adapted for the K–12 public school district setting as well as to pre-emptively identify barriers to implementation. Input about successful programs from other similar settings were used to develop and iteratively adapt an operational plan that included feedback from local stakeholders and barriers within the public school district.

#### Iterative process for refining the program

**Fig. 1.**
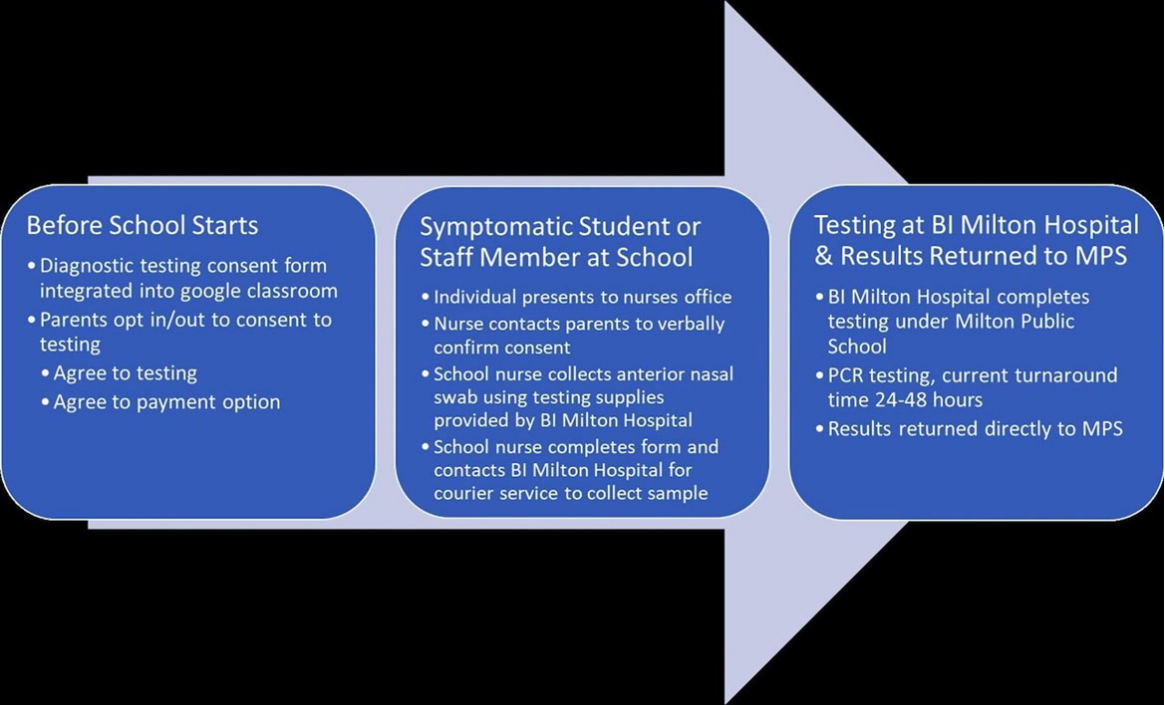
Process map of the initial proposed testing process.

First, community stakeholders were identified, and opinions were collected about school system needs and potential solutions. After identification of a potential local testing site and discussions about the potential for a public–private partnership to provide diagnostic testing services, a pilot process was developed. The proposed plan was then shared with key stakeholders, who were able to provide input and feedback. Thereafter, an updated plan was developed and the process restarted. After several cycles of feedback, the final plan was presented. An overview of the initial proposal is presented in Figure 1.

#### Initial program elements and development process

After identification of a need to improve access to COVID-19 diagnostic testing to support school reopening, different avenues for provision of testing services were explored, including public–private partnerships and integration of testing into town services. As part of these discussions, the superintendent of the school system identified the president of the local community hospital as a potential partner. The president had previously reached out the school district and offered to support the public school system with the reopening process. Other options (eg, partnering with a local college with a testing plan, testing provided by the town, partnering with a private for-profit testing company) were deemed infeasible and were not pursued. The decision was made to move forward with a public–private partnership testing delivery model, and representatives from the health subcommittee met with key members of hospital leadership. They offered a version of the testing plan that had been proposed to a local community college, adapted for public school use.

#### Elements of the initial proposal

The initial iteration of the proposal presented for the public school system included the following elements: (1) polymerase chain reaction (PCR) testing available to symptomatic students and staff; (2) testing free of charge to public school teachers and staff returning for in-person educational activities; (3) testing provided at-cost to students after scaling up; (4) installation of a high-throughput testing assay slated to be available in late fall 2020; and (5) providing test results directly back to the public school system through a new account embedded within their system. These results could also be provided to the local board of health for rapid reporting purposes. Less invasive anterior nares samples would be collected by school nurses using kits provided by the community hospital. Critically, in the initial proposal, the hospital offered to provide a courier for collecting samples from the individual schools where the testing would be performed on site as well as a fee waiver for teachers. To ensure that test results were received by the school system, the hospital offered to create an account for the school system where results would be entered and provided. Initially, the hospital proposed faxing results to a monitored machine.

#### Integration into system and pretesting planning

To ensure a short turnaround time from identification of a need for a diagnostic test to collection, relevant forms, including consent forms, screening forms, and scheduling forms, were integrated into the school’s virtual school classroom. These forms could be completed and then transmitted using a secure system to transmit relevant protected health information. Importantly, because the request and the order were to be placed within the school’s platform, a record of the test order was created that the school could use to track results among those who opted into the program. In parallel, a system for ordering and scheduling testing under the public school banner was created in the local community hospital system.

#### Process for collecting the sample and receiving the results

In considering the implementation, barriers to testing access and the need for a closed system were major concerns raised by key community stakeholders, including those serving on the town health committee. Thus, the plan for sample collection and returning results was planned to be fully embedded into the school system. This strategy limited concerns about other barriers (eg, transportation) and ensured that results would be received within the school system, such that they could be acted upon expediently. Specifics of the process are presented in Table 2. After tests were completed, the results would either be sent via secure fax or sent via encrypted e-mail to the school system. Once received, the school system would then provide the results back to the school staff member or student who requested the diagnostic testing.

**Table 2.** Stepwise Sample Collection Process Embedded within the School Infrastructure

1. School nurse screens individual for symptoms. 2. If the individual meets criteria for testing within district guidelines, then the school nurse confirms verbal consent with the student’s parents or guardian, or with the school district staff member, for testing. 3. After obtaining/confirming verbal consent for testing, the school nurse will obtain an anterior nares swab using testing materials provided by the hospital. a. Anterior nares sample collection is not an aerosol-generating procedure and thus does not require specialized personal protective equipment (PPE) by the nurse, or specialized ventilation systems. b. Nurses collecting sample will be instructed to wear the same PPE as is provided in the healthcare setting for collection of these samples (surgical mask plus eye protection) though they have the option of using an N-95 mask provided by the district, if they prefer. 4. Form provided by the hospital completed, serves as an order form for testing and indicates to whom in the school district the test result is returned. 5. Courier contacted, who will come to the school location with the symptomatic individual and collect the sample. 6. The hospitall will conduct PCR testing, with a current turnaround of 24–48 hours. This result will be reported by the laboratory to the town and the state as required by law.

#### Changes based on stakeholder feedback

**Fig. 2.**
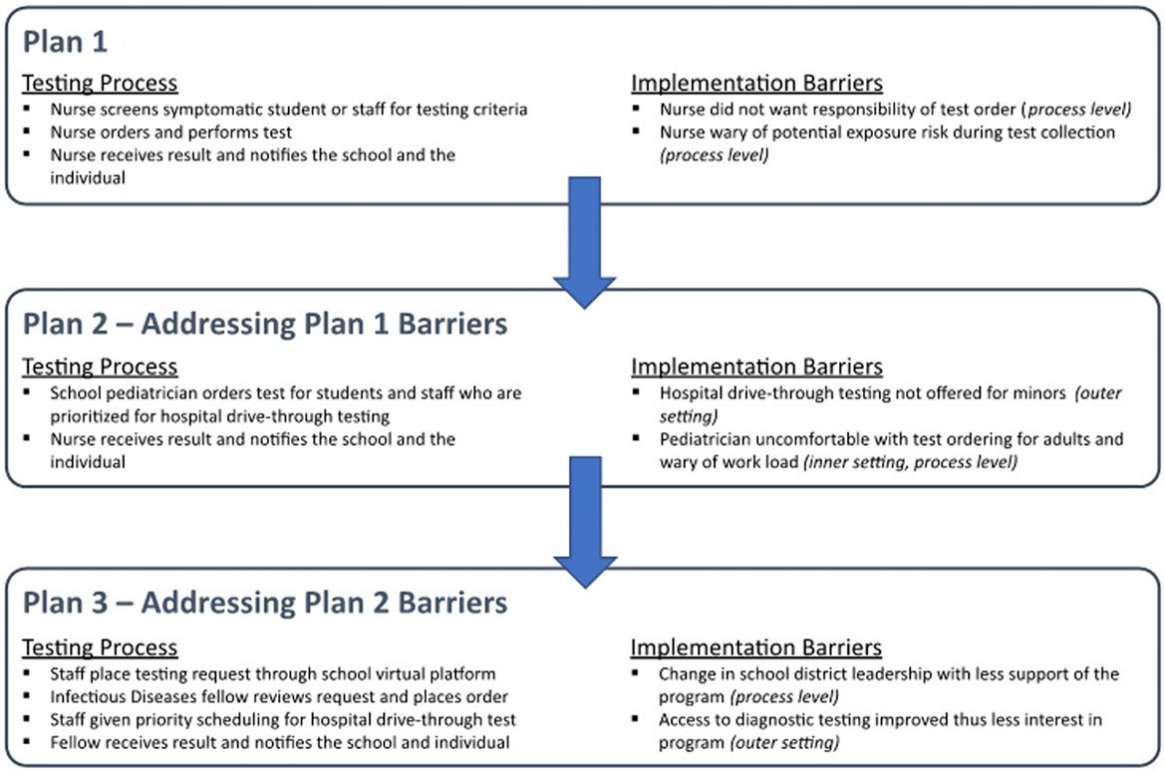
Iterative changes to the testing process based on implementation barriers.

Stakeholders in the school system identified several barriers and challenges with the initial proposal. Iterative changes to the plan’s testing process (ie, how they were addressed in subsequent plan revisions) are presented in Figure 2. Specifically, school nurses expressed concerns about limited staff time to collect samples, safety concerns about transmission of infection during sample collection, and concerns that ordering and receiving SARS-CoV-2 results was outside their scope of practice, potentially exposing them to legal jeopardy. Based on this feedback, several adaptions were proposed to improve feasibility and acceptability of the testing program at the process and inner setting levels. Specific changes included requesting that the order be placed by the part-time school physician (a pediatrician) and provided back to the school system. To eliminate the need for nurses to collect samples at the schools themselves, the local hospital offered to provide priority scheduling to school staff for sample collection using their drive-thru testing program; however, the hospital could not provide testing services to students given hospital age restrictions. Other elements of the plan, including the closed-loop communication and the waiver of testing fees, were maintained.

#### Additional feedback and adaptation

A revised proposal was then presented. However, stakeholders continued to express concerns about scope of practice, legal liability, and time constraints associated with the part-time school pediatrician ordering and following up test results for students and adult staff. Additionally, concerns were expressed about faxes being sent to a central location, thus potentially leading to challenges with Health Insurance Portability and Accountability Act (HIPAA) compliance and about the fax machine not being monitored during weekends and holidays, potentially leading to loss of follow-up and delays in communication of results. Based on identification of these additional barriers, a collaboration was developed with the academic teaching affiliate of the public hospital. In this iteration of the proposal, a testing request form would be submitted through the online classroom portal and transmitted electronically to clinical infectious diseases fellows at the academic affiliate, who would be compensated for their time with funds from the public school district. To ensure that results would be communicated back to the public school district for infection prevention purposes and to make decisions about need for quarantine and follow-up, the form included consent to release of results back to the public school district. The clinical infectious diseases fellow on call for the week would then review the request and place the order in the electronic ordering system. After the order was placed, a notification was provided to the school staff member requesting the test, who was then able to call a dedicated scheduling line to arrange for an appointment at the drive-thru testing center. Test results were then provided back to the ordering clinical fellow, who would then contact the requesting individual and provide the results and any necessary counseling and would also report results back to the school district for review.

#### Reasons for failure to progress from preimplementation to implementation

Ultimately, proposal for an integrated diagnostic testing plan, which involved a multilevel partnership, did not move from the preimplementation stage to full implementation. The failure was primarily driven by factors that can be traced to the inner and outer settings of CFIR, although process elements and complexity of the intervention also played a role.

#### Major determinants

First, at the time this program was being developed in the summer of 2020, the lack of a published precedent or model for providing testing within a single public school district created major challenges moving the program forward. Multiple stakeholders were concerned about liability (external construct). Other reasons for failure were traced to the CFIR inner setting, specifically, changes in public school leadership. A key champion of promoting and moving the testing program forward was the school district superintendent. Support of this individual helped with the development of the public–private partnership and engagement of stakeholders whose support was necessary for the operation of the program. Shortly before the start of the school year, and while the process was being developed, this individual resigned unexpectedly and was replaced with an acting superintendent. The newer leadership had weaker connections to the local hospital and was less supportive of pilot testing an integrated testing program. Thus, the loss of a critical champion at the inner setting was a major determinant of the program’s failure to progress from the pre-implementation stage to implementation.

#### Additional factors

In addition to the 2 major determinants of failure, the gradual improvement in widespread access to testing (external construct) created a perception that the program was no longer necessary, reducing local support (inner construct). The complexity of the intervention, requiring a public–private academic partnership combined with a lack of a framework and experience with the topic within the public school system also contributed to failure (ie, intervention construct). Failure to gain support from key stakeholders, specifically, school nursing staff, was a persistent barrier (ie, process and inner setting). The strategy identified to solve this problem—an academic partnership—created additional complexities at the level of the intervention, which in turn contributed to failure to progress from preimplementation to implementation. At the process level, other opinion leaders in the public school system remained unsupportive of the concept of integrating testing, perceived as a medical intervention, into a school setting. Stakeholders were uncomfortable with the proposal that the schools would become involved with testing in any way (ie, inner construct). The response was to iteratively update the proposal based to address concerns by shifting the responsibility of ordering and communicating test results to physicians at the local hospital. Although discomfort was partially alleviated by the final proposal, this innovation occurred too late for sufficient support to move the program forward.

## Discussion

We constructed an iterative preimplementation process for developing a COVID-19 diagnostic testing program integrated within a public school system. Although our local diagnostic testing program did not progress to the implementation phase, identification of key barriers within different CFIR domains to inform reasons for lack of progression may be useful for others attempting to embed a diagnostic testing program within public schools. The testing program was ultimately unsuccessful due to challenges at most CFIR levels but particularly the inner and outer settings.

The first superintendent viewed creation of a novel and potentially scalable program as a solvable challenge that offered an opportunity to provide a long-term contribution to public education; however, the superintendent’s replacement did not place the same priority on innovating in-school testing programs. Lack of a clear, publicly available precedent and paradigm for conducting and pilot testing such a program within the public school system was a major barrier.

Although we sought to identify and include all relevant stakeholders at the outset of program development, multiple individuals with concerns continued to manifest throughout the process. In the future, novel programs should be highlighted at open meetings at the inception of development to ensure that concerns can be addressed and potenital solutions identified from the beginning of the process.

Improved access to testing in the community rendered the urgency of the program apparently less critical; however, increased access did not solve the problem of a lack of a closed-loop communication process or improve logistical challenges this testing program design sought to address.

Over the course of the academic year, COVID-19 screening using antigen tests became available in Massachusetts public school settings due to state-wide support with infrastructure and funds.^3–5^ However, screening programs do not provide testing services to symptomatic individuals. At the time of development of our pilot diagnostic testing program, no such program had been integrated into schools for public school students and staff in the United States.

Fortunately, prior to the 2021–2022 academic school year, availability of both in-school COVID-19 diagnostic and screening testing dramatically increased across the country. In Massachusetts specifically, districts have been strongly recommended to participate in both the diagnostic and screening pooled testing components of the COVID-19 testing program. Rapid diagnostic testing is available for symptomatic individuals at the point-of-care through a state-funded and supported program. The testing programs are managed at the state rather than the local level, and they include external staff support, funding, and provision of testing supplies. Higher-level state support as well as allocation of federal funds were critical for overcoming the barriers identified at the local level and moving in-school testing from the preimplementation phase to implementation.

Systems and processes refined during the creation of this diagnostic testing program may be applied to future years to support districts still developing their diagnostic testing programs for COVID-19, as well as other respiratory viruses, including influenza.^14^ Broader diagnostic testing of respiratory viruses may be important as infection control strategies, such as masking and distancing, are relaxed and as the prevalence of other respiratory viruses with similar clinical syndromes rises,^15^ creating challenges for school districts if diagnostic test results are not rapidly available. Access to a point-of-care, multivirus diagnostic test^14^ within the schools could facilitate decision making about the need for school closures, quarantines, or widespread testing of students and staff.

In conclusion, we developed a roadmap and process for implementing a novel COVID-19 diagnostic testing program embedded within a public school system through a proposed innovative public–private partnership. Ultimately the program failed for several reasons; however, lessons learned about types of barriers and potential solutions could be leveraged in the future to support similar partnerships to support essential services not previously applied to school settings.
